# Anti-Oxidant, Anti-Hemolytic Effects of *Crataegus aronia* Leaves and Its Anti- Proliferative Effect Enhance Cisplatin Cytotoxicity in A549 Human Lung Cancer Cell Line

**DOI:** 10.31557/APJCP.2020.21.10.2993

**Published:** 2020-10

**Authors:** Islam Omairi, Firas Kobeissy, Salam Nasreddine

**Affiliations:** 1 *Doctoral School of Science and Technology, Research Platform for Environmental Science (PRASE), Faculty of Sciences, Lebanese University, Lebanon. *; 2 *Department of Biochemistry and Molecular Genetics, Faculty of Medicine, American University of Beirut, Beirut, Lebanon, Lebanon. *; 3 *Department of Psychiatry, Center for Neuroproteomics and Biomarkers Research, University of Florida, Gainesville, FL, USA.*; 4 *Department of Biology, Faculty of Sciences-Section I, Lebanese University, Groupe Anti-Cancer Therapeutic Approaches (ATAC), Laboratory Rammal Rammal, Lebanon. *

**Keywords:** Crataegus aronia, antioxidant activity, antiproliferative activity, cells migration, cells aggregation

## Abstract

**Objective:**

For Arabian traditional medicine, *Crataegus aronia* syn. Azarolus (L) Bosc. ex DC (Rosaceae) is widely used to treat diabetes, sexual weakness, cardiovascular diseases and cancer. The anti-cancerous and anti-hemolysis effects of the hydroalcoholic extract of this plant have never been investigated before. The present study aims to evaluate the biological activities of the hydroalcoholic extract of *Crataegus aronia* leaves in combination with cisplatin, one of the most widely employed chemotherapeutics, on A549 human lung cancer cell line.

**Methods:**

The anti-oxidant and anti-proliferative activities of leaves, fruits, seeds of *C. aronia* were investigated by DPPH method and MTT assay; respectively. Cell migration activity was investigated by wound healing and by cell aggregation assays. The effect of *C. aronia* in inducing cell cycle arrest along with activating cell apoptosis was evaluated by flow cytometry and Western blot assays, respectively.

**Results:**

Our results showed that *C. aronia* leaves (*C. aronia* L.) had the highest anti-oxidant and anti-proliferative activities. The leaves extract was potent against hemolysis of the human erythrocytes and showed elevated decrease in migration by reducing wound healing migration and by increasing cell aggregation. Finally, *C. aronia* L. treatment exhibited apoptotic activity on A549 cells by the down-regulation of PARP-1, caspase-3 and Bcl-2 proteins and by increasing the percentage of A549 cells in sub G0 cell cycle. Moreover, the co-treatment of *C. aronia* L. and cisplatin remarkably sensitised A549 cells to cisplatin.

**Conclusion:**

The results suggested that *C. aronia* L. could be used as a potential treatment against human lung cancer exhibiting minimal side effects on human health.

## Introduction

Cancer is considered the second leading cause of death worldwide accounting for an estimated 18.1 million new cancer cases with around 9.6 million cancer deaths in 2018 (Bray et al., 2018). It is estimated that cancer-related deaths would increase to over 17 million in 2030 (Thun et al., 2009). Cancer diagnosis, death and treatments are strongly varied among countries depending on the environmental factors, economic development and lifestyle factors. Lung cancer is considered a major cause of cancer diagnosis (11.6% of the total cases) and deaths (18.4% of cancer deaths) in both sexes combined (Bray et al., 2018). Lung cancer is mainly divided into two key types involving the non-small cell lung cancer (NSCLC) and the small cell lung cancer (SCLC). NSCLC is divided into 3 major subtypes: squamous cell carcinoma, adenocarcinoma and large cell carcinoma. It accounts for 75 % of all lung cancer cases.

Small cell lung cancer (SCLC) accounts for 15 % to 18 % of all lung cancer cases (Miao et al., 2018). Tobacco smoking remains the number one risk factor for developing lung cancer. In the United States, lung cancer mortality rate, caused by cigarette smoking, ranges between 80% to 90% (Alberg et al., 2013).

The chemotherapy is the classical treatment for lung cancer and often consists of a combination of drugs. The most common chemical drugs used in lung cancer treatment are cisplatin, gemcitabine, paclitaxel, carboplatin plus docetaxel (Burdett et al., 2008). Since chemotherapy has serious side effects, herbal medicine was used to increase the efficacy of chemotherapy drugs and to reduce their side effects as well as to prolong the patient’s survival. In China, more than 133 herbal supplements have been historically used to treat lung cancer. The most common herbal treatment used alongside chemotherapy for lung cancer are astragalus, Nan Sha Shen and asparagus roots (Jiao et al., 2017). However, in the United States, plant-derived drugs represent about 25% of the total drugs, compared to countries like India and China where the contribution is ~80% (Qazi Majaz and Molvi Khurshid, 2016).

In fact, there is a return to traditional herbal medicine to treat different diseases and especially cancer. Since the Mediterranean region is very rich in medicinal plants, Lebanon is considered a promising country for new plant discoveries (Baydoun et al., 2015). The *Crataegus aronia *syn. Azarolus (L) belongs to the family of Rosaceae; it is one of the most dominant hawthorn species that populated the mountains of the Mediterranean region and has been utilized in traditional medicine against many diseases like sexual weakness, cardiovascular diseases, diabetes and cancer (Ali-Shtayeh et al., 2000; Ljubuncic et al., 2005). Several studies have shown that the high amount of flavonoids and proanthocyanidins in the genus *Crataegus* are very effective against cardiovascular problems, such as hypercholesterolaemia, hypertension, myocardial injuries and atherosclerosis (Nabavi et al., 2015). In addition, *C. aronia* also has showed to have anti-obesity and hyperglycemic effects (Al-Hallaq et al., 2013). Many phytochemical studies revealed that the *Crataegus aronia* are rich in phenolic compounds including flavonoids, phenolic and tannins (Amina et al., 2018; Donno et al., 2017), which are known to have anti-inflammatory (Brezani et al., 2018), anti-carcinogenic and anti-antioxidant activities (Newmark, 1996; Pourreza, 2013). However, despite its wide use in traditional medicine, the literature still lacks scientific pieces of evidence about its anticancer effects. In the present study, we aimed to determine the antioxidant and the anti-hemolytic activity of hydroalcoholic extract of *C. aronia* and to evaluate its anti-migration, anti-invasion and anti-aggregation effects on the human lung adenocarcinoma cell line (A549). Also, we aim to evaluated the anticancer activity of cisplatin in combination with *C. aronia* plant extract. Our data are the first to highlight the anti-cancerous activity of *C. aronia* on human lung adenocarcinoma cell line.

## Materials and Methods

*Plant collection and extraction*

Fresh *C. aronia* plant was collected Almat, Byblos, Mount Lebanon between May 2018 and Jun 2018. A voucher specimen (ul 111) was stored in the hebanium of the Faculty of pharmacy, Lebanese University, Beirut, Lebanon. Professor George TOHME, the herbalist and president of the National Council for Scientific Research (CNRS)-Lebanon, carried out the botanical authentication. Leaves, yellow fruits and seeds of *C. aronia* that were processed. In summary dried plant parts were ground to powder and kept in plastic containers and kept away from light, heat and moisture till use. After that, 10 g of *C. aronia* powder plant was kept in 200 ml conical flask to which 100 ml of 70% ethanol was added. The flasks were kept in a reciprocating shaker for 3 to 4 days for continues agitation at 150 rev/min. The solutions were then filtered and concentrated by rotary evaporator at 40°C and lyophilisator, respectively and stored at -20°C. 

*Cells and Cell Culture*

Human lung adenocarcinoma A549 cell line was purchased from the American Type Culture Collection (ATCC, Manassas, VA). A549 cell line was maintained in Dulbecco’s Modified Eagle’s Medium (DMEM) (Sigma-Aldrich Co., D0819) with 10% Fetal Bovine Serum (FBS) (Sigma-Aldrich Co., F9665) and 100U/ml penicillin and 0.1 mg/ml streptomycin (Sigma-Aldrich Co., P4333). Cells were incubated at 37°C in humidified air containing 5% CO_2_.

*DPPH (α, α-diphenyl-β-picrylhydrazyl) antioxidant assay*

To assess the antioxidant activity of *C. aronia* plant extracts (leaves, fruits and seeds), DPPH radical scavenging activity was performed according to a previous method (Nasreddine et al., 2018). DPPH (Alfa Aesar 44150) is dark-colored crystalline powder-free radical that gives a purple color when dissolved in a solvent. It works by trapping or scavenging other free radicals present in the solution, which will cause the change of the purple color into a pale-yellow color as an indication of the antioxidant activity of the compound tested. Here, 1 ml of each concentration of the plant extract was prepared to which 1 ml of DPPH was added. The control was prepared with 1 ml of DPPH and 1 ml of solvent (ethanol). Samples were kept in the dark for 30 min and the optical density was measured at 515 nm wavelength with UV-Vis spectrophotometer. The DPPH scavenging activity of the samples was calculated according to the following equation: 

% Radical scavenging activity = [(Abs control − Abs sample)]/ (Abs control)] X 100.

*MTT (3-(4,5-Dimethylthiazol-2-yl)-2,5-Diphenyl Tetrazolium Bromide) assay*

A549 cell lines were seeded in a 96-well plate with 10^4^ cells per well in 200 µl DMEM culture medium with 10% FBS and 1% (Pen/Strep). After incubation overnight, cells were treated with different concentrations of *C. aronia* extract (50, 100, 150, 200, 300 and 500 µg/ml) for 24, 48 and 72 hr. After incubation time with treatments, 20 µl of MTT (3-(4,5-Dimethylthiazol-2-yl)-2,5-Diphenyl Tetrazolium Bromide) (Sigma-Aldrich Co., M5655) (5 mg/ml in PBS 1X) reagent is added to each well and left for 3 hr at 37°C with 5% CO_2_ until a purple precipitate was observed. The MTT was dissolved in 1X phosphate-buffered saline (PBS) (Sigma-Aldrich Co., D1408). Media was removed delicately and 100 µl isopropanol-HCl (MTT solvent) was added to dissolve the purple precipitate (formazan crystals). The resulting purple formazan crystals were dissolved in 100 µl isopropanol-HCl. The Optical Density (OD) was read by an ELISA reader at 595 nm wavelength. Isopropanol-HCl was used as a blank. Percentage of cell viability was calculated according to the following equation: 

Cell viability (%) = (OD treated/ OD control) *100. Percentage cytotoxicity was calculated for finding the IC50 value of the concentration required for 50% cell death by each extract.

*Wound healing assay*

Wound healing assay was done to determine the ability of A549 cells to migrate after the treatment with different non-toxic concentrations of *C. aronia* by measuring the distance of the wound formed. Briefly, 7 x 10^5^ cells were seeded in a 6-well plate with DMEM medium (10% FBS and 1% Pen/Strep) and incubate overnight at 37°C to reach 90-100% confluency. After incubation time, the A549 cells were starved in DMEM culture medium without FBS. Using a 200 µl tip, a scratch was made as uniformly as possible through the monolayer cells. After that, treatment of different concentrations (150 and 300 µg/ml) of leaves of *C. aronia* was added. Representative pictures were taken at different time points (0, 2, 4, 6, 8, 10, 12 and 24 hr) using Leica DM IL LED inverted microscope (Wetzlar, Germany Leica Microsystems GmbH) equipped with digital imaging system. The distance of the wound at different time points was measured using ImageJ. The results at each time point were compared to t=0 (wound distance at t=0 – wound distance at different time points) and the results were further compared to the control with no treatment, to estimate the migratory potential of the cells. In this technique, the concentrations of the plant extracts used are tested before by MTT assay to be non-toxic to the cells with the selected incubation time.

*Aggregation assay*

The aggregation assay was performed according to the published protocol (Al Dhaheri et al., 2013). A549 cells were seeded in a 6-well plate and detached using 2mM EDTA in calcium magnesium-free PBS (CMF-PBS). A cell suspension of 1 x 10^6^ cells/ml was aliquoted into centrifuge tubes and washed with CMF-PBS. Cells were then resuspended in 1ml CMF-PBS with or without leaves of *C. aronia* (150 and 300 μg/ml) and kept on a rocker for 60 minutes at 37°C. Cells were fixed with 1ml of 1% formaldehyde and pictures were taken under an inverted microscope (Leica Microsystems GmbH, Wetzlar, Germany). Percentage of aggregation was calculated using the following equation: 

% Aggregation= [(1-Nt/Nc)] X 100, where Nt and Nc represent the number of single cells in treated or untreated (control) groups, respectively. 

*Flow cytometry and cell cycle analysis*

A549 cells were seeded in a 6-well plate with a density of 2.8x10^5^ cells per well and incubated overnight until 70% confluency is reached. Post 24 hr incubation, cells were treated with 150 and 300 µg/ml of leave extracts of *C. aronia* and incubated for 48 hr. Then, cells were removed and fixed with 1ml of ice-cold 70% ethanol and kept at 4°C until analysis. Fixed cells were washed two times in PBS 1X and collected by centrifugation before being resuspended in 50 μl of 100 µg/ml of RNase A (Sigma-Aldrich Co., R6513) and incubated for 1 hr at 37°C. Cells were resuspended in 200 µl of 50 µg/ml of Propidium Iodide (PI) (Sigma-Aldrich Co., 81845). Cell cycle analysis was determined using the BD FACS Aria cell sorter machine (Guava EasyCyte8 Flow Cytometer; Millipor).

*Hemolytic activity*

Hemolytic activity was done according to the protocol of Malagoli, (2007). Briefly, fresh human blood sample was centrifuged at 2500 rpm for 10 min to separate erythrocytes from the plasma. Erythrocytes were washed three times with 1X PBS. Discard the supernatant by centrifugation at 2,500 rpm for 12 min at 4°C. The erythrocytes were then diluted with 1X PBS to obtain a suspension of 5%. To 1 ml of the erythrocyte suspension, 50 µl of different concentrations of leaves of *C. aronia* extracts were added (10, 20, 40, 50 and 100 µg/ml). The mixture was incubated at 37°C for 1 hr and a half and then the tubes were centrifuged at 2,500 rpm for 10 min at 4°C. The optical density (OD) was then measured at 540 nm. Blood alone without treatment was used as negative control, SDS 1% to check hemolysis of cells and PBS 1X as the blank. Hemolytic levels were calculated as follows:

% Hemolysis = [OD of extract / OD of control] X100.

*Anti-hemolytic activity*

For the anti-hemolytic activity assessment, we followed published protocol for anti-hemolytic activity (James and Alewo, 2014). Same steps were performed as the hemolysis assay except adding of H_2_O_2_ to induce hemolysis. Fresh human blood sample was centrifuged at 2,500 rpm for 10 min to separate erythrocytes from the plasma. Erythrocytes were washed three times with 1X PBS by centrifugation at 2,500 rpm for 12 min at 4°C and the supernatant was discarded. The erythrocytes were then diluted with PBS to obtain a suspension of 5%. To 1 ml of the erythrocyte suspension, 50 µl of different concentrations of leaves of *C. aronia* extracts were added (10, 20, 40, 50 and 100 µg/ml). The mixture was incubated for 20 min with the treatment and then 350 µl of H_2_O_2_ was added. The mixture was incubated at 37°C for 1 hr and a half and then the tubes were centrifuged at 2500 rpm for 10 min at 4°C. The optical density (OD) was then measured at 540 nm. Blood alone was used as negative control, H_2_O_2 _30% alone as positive control and PBS 1X as the blank. Anti-hemolytic levels were calculated according to the following equation:

% of inhibition of hemolysis = [(OD of control – OD of extract) / (OD of control)] X 100.

*Western blot*

A549 cells were seeded in a 6-well plate with a density of 2.8x10^5^ cells per well and incubated overnight. After 24 hr, leaves of *C. aronia* (150 and 300 µg/ml) extract were added to the wells and incubated for 48 hr. Then, cells were lysed 120 µl of lysis buffer (Tris-HCl pH=6.8, glycerol, 10% SDS, DTT and protease inhibitor) for 20 min at 4°C. After centrifugation, the supernatant was collected and stored at -20°C until use. Protein quantification was done according to Bradford assay. A total of 20 µg of protein samples were mixed with Laemli and β-mercaptoethanol and denatured at 95°C for 10 min. Then, 20 µl of each sample were loaded to a 10% SDS-PAGE gel. Proteins were then transferred to a nitrocellulose membrane for 2 hr. The membrane was then fixed with 5% non-fat dry milk for 2 hr and incubated with rabbit primary antibodies for 2 hr or overnight. All the primary antibodies are Rabbit IgG anti-human: poly-adenosine diphosphate (ADP) ribose polymerase-1 (PARP-1) (Cell Signaling, 9542) (dilution 1/1,000), caspase-3 (Santa Cruz, sc-7148) (Dilution 1/1,000), B-cell lymphoma 2 (Bcl-2) (Abcam, ab32124) (dilution 1/5000), Bcl-2 associated X protein (Bax) (Abcam, ab32503) (dilution 1/2,000) and GAPDH (Abcam, ab181602) (dilution 1/10,000). The secondary antibody Horseradish Peroxidase (HRP)-conjugated Goat anti-rabbit IgG (Abcam, ab6721) (dilution 1/5,000) was used to detect the proteins of interest. The bound secondary antibody was detected with ECL Plus kit according to the manufacturer’s protocol. The autoradiographs obtained were scanned using the Chemidoc imaging system (Bio-Rad) and the intensity level of the bands was quantified using Image J software 1.48 (National Institutes of Health, Bethesda, MD, USA). All bands were normalized to GAPDH loading control. 

*Statistical analysis*

Data were expressed as mean ± standard error of the mean (SEM). The difference between two groups was determined by One-way Anova followed by Bonferroni test to calculate values (p). All statistical analyses were performed using Graphpad Prism 8 software (GraphPad Software Inc., San Diego, CA, USA). A value of *p< 0.05, **p < 0.01 and ***p< 0.001 were considered to indicate statistical significance.

## Results

*Antioxidant activity of different parts of C. aronia* *using DPPH radical scavenging assay*

**Figure 1:**
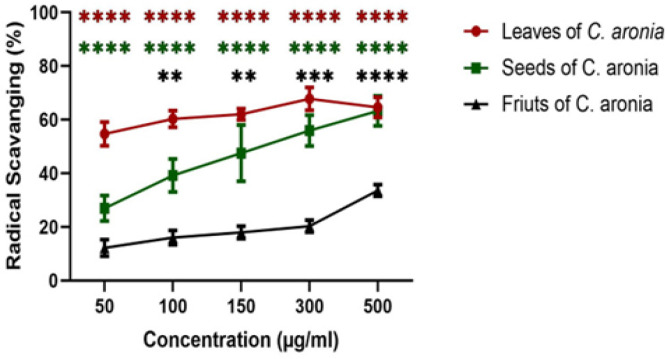
Antioxidant Activities of Ethanol Extracts of *C. aronia.* The percentages of radical scavenging activity of different concentrations (50, 100, 150, 300 and 500 µg/ml) of leaves, seeds and fruits of *C. aronia* plant extracts were measured by DPPH radical scavenging assay. Data are presented as the mean ± SEM for three independent experiments (**p<0.01, ***p<0.001, ****p<0.0001)

In order to test the antioxidant activity of *C. aronia, *DPPH radical scavenging activity was performed as previously mentioned and radical scavenging percentage of each plant part (leaves, seeds and fruits) extract was calculated. The antioxidant activity of the plant extract is considered high when more DPPH radicals (DPPH·) are neutralized and transformed into DPPHH leading to a change in the solution color from dark-violet into pale-yellow or colorless solution. Lower absorbance of the reaction mixture indicated by an increase in the radical scavenging percentage and; thus, higher antioxidant activity. In this study, the results showed an increase in radical scavenging percentage with the increase in the concentration of *C. aronia* extracts (50, 100, 150, 300 and 500 µg/ml). All the parts of *C. aronia* (leaves, seeds and fruits) showed a significant increase in the radical scavenging activity, with the highest percentage of antioxidant capacity in the leaves of *C. aronia* reaching 70% of free radical scavenging activity at 500 µg/ml with an IC_50_ of 14.66 ±1.166 µg/ml (Figure 1). This potent antioxidant activity of *C. aronia* leaves (*C. aronia* L.) could be attributed to the presence of phenolic compounds and can have therapeutic implications in the future to protect cells and tissues against oxidative damage (Huang et al., 2017).

*C. aronia* *plant extract reduce cell viability of lung cancer cell line*

**Figure 2:**
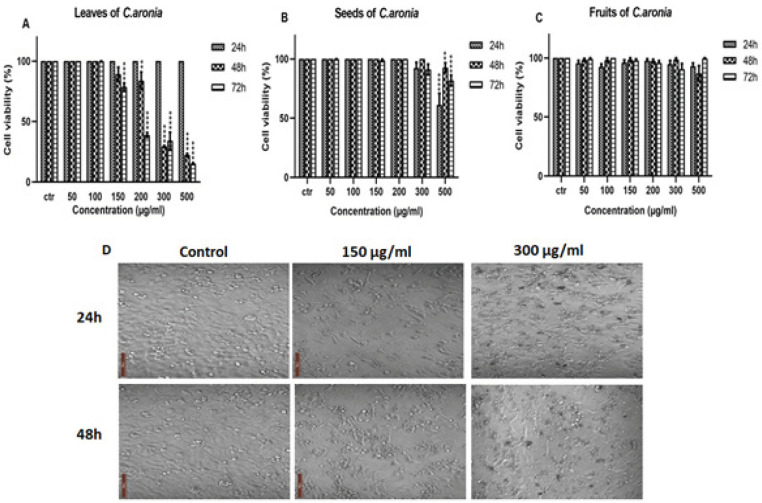
Cell Viability of A549 Lung Cancer Cell Line after *C. aronia* Treatment was Evaluated by the MTT Assay. (A-C) A549 cells were treated with the indicated concentrations of leaves (A), seeds (B) and fruits (C) of *C. aronia* ethanol extracts for 24, 48 and 72 hr. (D). Microscope view of A549 cells after 24 and 48 hr of treatment with leaves of* C. aronia* (150 and 300 µg/ml). The morphology of A549 cells was visualized under light microscope (magnification, x400). Data are presented as the mean ± SEM for three independent experiments (**p<0.01, ****p<0.0001)

A549 cell line was treated with increasing concentrations of different parts of *C. aronia* (leaves, seeds and fruits) for 24, 48 and 72 hr. MTT experiment was done as previously described and cell viability was assessed according to the formula mentioned above. Cell morphology and the number of A549 cells after their treatment are presented in Figure 2. The results showed that the leaves of *C. aronia* decreased the cell viability of A549 significantly at 200, 300 and 500 µg/ml concentrations after 48 and 72 hr of treatment, whereas no decrease in cell viability was observed at lower concentrations. While for the seeds of *C. aronia*, there was a significant decrease in cell viability of A549 cell line only at 500 µg/ml of treatment at all time points and no decrease at all in cell viability was observed in fruits of *C. aronia* (Figure 2 A, B and C). This indicates that *C. aronia* has a dose and time-dependent effect on the viability of A549 cells. In order to determine the concentration required to achieve a 50% inhibition of A549 cells induced by *C. aronia*, the dose-response curve was plotted. The IC_50_ values of *C. aronia* L. extract are 259**±**2.41 µg/ml and 195**±**2.29 µg/ml at 48 and 72 hr, respectively. According to the results obtained, the leaves of *C. aronia* (150 and 300 µg/ml), which have the highest anti-proliferative activity, were chosen for further experiments on A549 cell line. 

*Cisplatin reduces cell viability in lung cancer*

**Figure 3:**
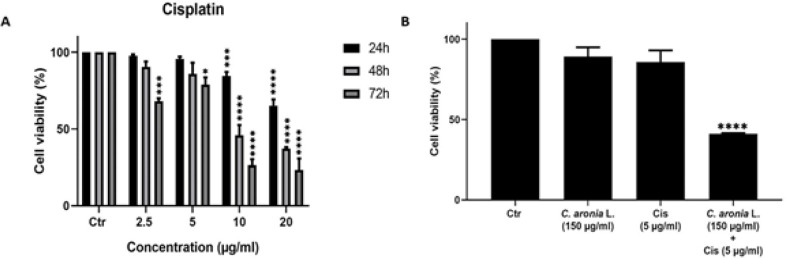
Cell Viability of A549 Lung Cancer Cell Line after *C. aronia* L. extract and/or Cisplatin Treatment was Evaluated by the MTT Assay. (A) A549 cells were treated with different concentrations of cisplatin (2.5, 5, 10 and 20 µg/ml) for 24, 48 and 72 hr. (B) A549 cells were treated for 48 hr with the indicated concentrations of cisplatin (Cis) and *C. aronia* leaves (*C. aronia L*.) separately and with combination of Cis and *C. aronia* L. Data are presented as the mean ± SEM for three independent experiments (*p< 0.05, ***p<0.001, ****p<0.0001)

Cisplatin (Cis) is a conventional chemotherapy used to treat various cancers (Dasari and Tchounwou, 2014), including lung cancer (Hotta et al., 2004). Cisplatin was used in many studies to induce an anti-proliferative effect against A549 lung cancer with an IC50 of 10 μM after treatment for 48 hr (Tseng et al., 2016). Our purpose was to see the effect of cisplatin in A549 cell line. A549 cell line was treated with different concentrations of cisplatin (2.5, 5, 10 and 20 μg/ml) at 24, 48 and 72 hr time points (Figure 3A). As shown in Figure 3A, cisplatin inhibited the proliferation of the lung cancer cell line in a concentration- and time-dependent manner. The results obtained showed that the treatment of A549 cell line with cisplatin have an IC50 of 24.73, 9.56 and 4.72 μg/ml at 24, 48 and 72 hr as shown in Figure 3A. We then used the recommended low concentration of cisplatin (5 μg/ml) for further trials in A549 cell line. These results led us to study the effect of cisplatin in combination with *C. aronia* L.

*Co-treatment with cisplatin/ C. aronia* *leaves has a synergistic inhibitory effect on A549 cell viability*

The literature lacks any study that provides the combinational effect of *C. aronia* plant extracts and cisplatin. Due to the anticancer effect of the *C. aronia* leaves on A549 cell line, we hypothesized that an additional effect might appear when combined with cisplatin. To determine whether the combination of cisplatin and *C. aronia* L. extract may have a more significant anti-cancer effect on A549 cell line than a single treatment, A549 was treated for 48 hr with a low-dose of cisplatin (5 μg/ml) in combination with 150 µg/ml of *C. aronia* leaves. These doses of cisplatin and plant extract were chosen based on MTT assay results that showed a low level of toxicity of these concentrations on A549 cell line. After 48 hr of A549 cells treatment with *C. aronia* L. extract (150 μg/ml) and cisplatin (5 μg/ml), the cell viability decreased to 89.15% and to 85.88%, respectively (Figure 3B). However, cell viability of A549 was dramatically decreased to 41.14% after co-treatment with cisplatin and *C. aronia* leaves (Figure 3B). These results showed that the leaves of *C. aronia* boosted the cytotoxic effect of cisplatin against A549 cells.

*Anti-hemolytic activity of C. aronia* *leaves extract in human red blood cells*

**Figure 4:**
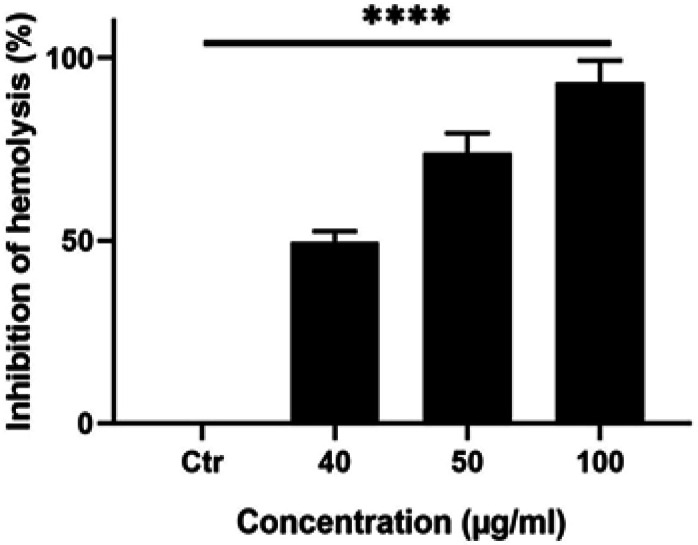
Effect of *C. aronia* Leaves Extract Against Hemolysis in Human Erythrocytes. The human erythrocytes were treated with different concentrations of *C. aronia* L. (40, 50 and 100 µg/ml) in the presence of H2O2-induced hemolysis of erythrocytes. Data are presented as the mean ± SEM for three independent experiments (****p<0.0001)

As the typical route of drug administration is systemically by infusion, it is important to monitor if *C. aronia* L. extract has any effect on human Red Blood Cells (RBCs) hemolysis. To assess if the extract can induce hemolysis in human blood RBCs, hemolysis assay was conducted. The results showed that *C. aronia* L. extracts do not cause hemolysis for human RBCs (data not shown) and thus, anti-hemolysis assay can be performed. Anti-hemolysis assay was performed as previously described (James and Alewo, 2014); in brief fresh human blood samples were incubated with the extract for 20 min with *C. aronia* L. followed by the addition of H_2_O_2_, which is the agent that causes hemolysis. This is done to see if these extracts will inhibit the function of H_2_O_2_. Blood samples were incubated with 40, 50 and 100 µg/ml of *C. aronia* L. extract for 20 min after which H_2_O_2_ was added. The results showed than the percentage of inhibition of hemolysis increase with increasing concentrations of the *C. aronia* L. extract (Figure 4). The % of inhibition of hemolysis has been increased by 86.8 % at 100 μg/ml of the *C. aronia* L. extract (Figure 4). These results confirm that the *C. aronia* L. extract has an excellent anti-hemolysis effect in human RBCs.

*C. aronia* *leave extracts increase the aggregation of A549 cells*

**Figure 5:**
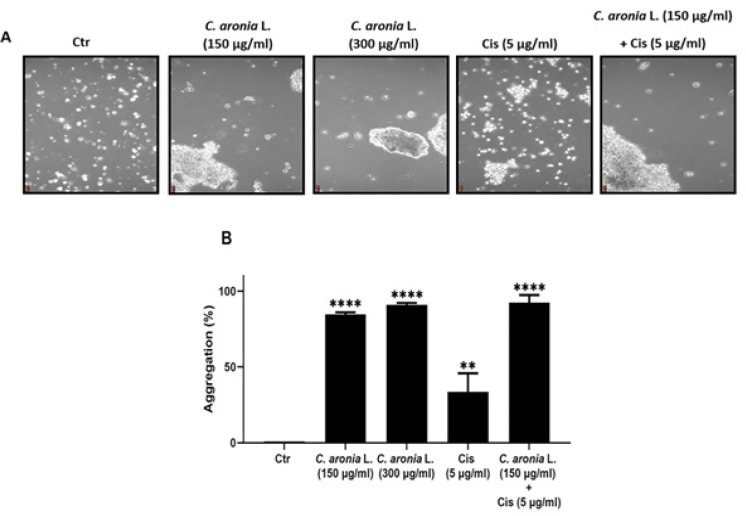
Effect of *C. aronia* L. in Aggregation of Human Lung Cancer Cell Line (A549). (A) A549 were treated for 1 hr with the indicated concentrations of Cis and *C. aronia* L. separately and with the combination of Cis and *C. aronia* L. A549 cells were photographed with light microscope at x100 magnification. (B) The histogram represents the percentage of aggregation of A549 cells in the presence of *C. aronia* L. extract and/or Cis. The % of aggregation is calculated as described in material and methods. Data are presented as the mean ± SEM for three independent experiments (**p<0.01, ****p<0.0001)

When cancer cells undergo metastasis, they lose their ability to adhere to each other so they can invade other organs (Leber and Efferth, 2009). When cancer is treated with chemotherapeutic drugs, the adhesion of cells should increase so the metastasis decreases (Larzabal et al., 2013). To determine whether our extract has an effect on the aggregation of these cells, aggregation assay was performed. Single cells number in control and in each treatment were assessed and aggregation percentage was calculated in each condition and compared to the control as previously described. After 1 hr of A549 cells treatment with 150 μg/ml and 300 μg/ml of *C. aronia* L. extracts, the percentages of cells aggregation increased to 84.67% and to 90.95%, respectively (Figure 5A, B). However, the percentage of cell aggregation of A549 is 33.61% after treatment with cisplatin alone (5 μg/ml) and become 92.40% after the co-treatment with cisplatin (5 μg/ml) and *C. aronia* L. (150 μg/ml) (Figure 5A, B). These results showed that the *C. aronia* L. extract dramatically increased the percentage of A549 lung cancer cells aggregation.

*C. aronia* *leave extracts decrease the migration ability of A549 cells*

**Figure 6:**
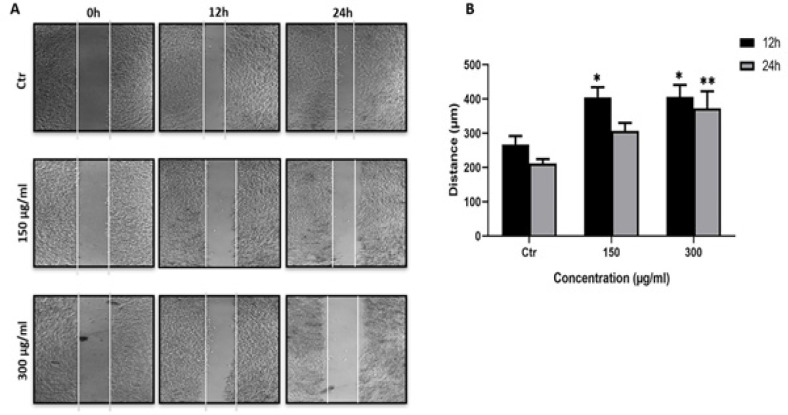
Effect of *C. aronia* Leaves Extract on A549 Cell Migration. (A) Wound healing assay of A549 cells treated with *C. aronia *L. (150 and 300 µg/ml) at 0, 12 and 24 hr after performing the scratch. A549 cells were photographed with light microscope at x40 magnification. (B) Histogram represents the distance (µm) that the A549 cells have migrated after treatments in 12 and 24 hr. Data are presented as the mean ± SEM for three independent experiments (*p< 0.05, **p<0.01)

Our previous results indicated that *C. aronia* L. extracts had an effect on cell aggregation, we hypothesized that these extracts would exhibit anti-metastasis effect. For this purpose, we performed the wound-healing migration assay with two non-toxic concentrations of *C. aronia* L. extract (150 μg/ml and 300 μg/ml) for 2, 4, 6, 8, 10, 12 and 24 hr. No detectable changes in distance were observed for cell migration within the first 10 hr (data not shown). As shown in Figure 6, the treatment of A549 cells with *C. aronia* L. extract significantly inhibited the cellular migration in concentration- and time-dependent manner. This data show that the *C. aronia* L. inhibits cellular migration of A549 cells. 

*C. aronia* *leave extracts induce apoptosis in A549 cells*

PARP-1, caspase-3, Bcl-2 and Bax proteins are considered as protein hallmarks of apoptosis. To check if the *C. aronia* L. induce A549 cell death after 48 hr of treatment via apoptotic machinery, we assessed the levels of PARP-1, caspase-3, Bcl-2 and Bax proteins by Western blotting analysis. 

**Figure 7:**
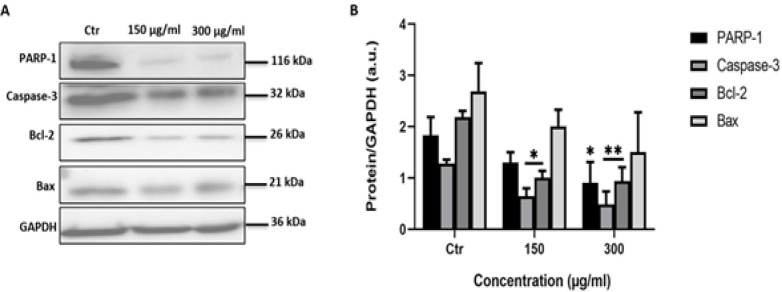
Effect of *C. aronia* L. on the Expression of PARP-1, Caspase-3, Bcl-2 and Bax Proteins in A549 Cells. (A) Representative western blot showing expression of target proteins in A549 cells exposed to *C. aronia* L. (150 and 300 µg/ml) extract. Levels of proteins were adjusted to the GAPDH protein (housekeeping gene). (B) The expression of proteins was normalized to the GAPDH, and the ratios were presented in arbitrary units (a.u.). Protein band intensity of target proteins was measured by ImageJ software. Data are presented as the mean ± SEM for 3 independent experiments

Poly (ADP-ribose) polymerase-1 (PARP-1) is a DNA-binding enzyme involved in DNA damage processing, genetic stability and apoptosis. During the execution phase of apoptosis, PARP-1 is specifically proteolyzed by caspase-3 (Amours et al., 2001). The activation of caspase-3 is a central event in the process of apoptosis (Thornberry and Lazebnik, 1998; Wolf and Green, 1999). Caspase-3 protein is proteolytically activated.When DNA is damaged in the cell, p53 expression will rise, leading to the increase in Bax protein expression, which is a pro-apoptotic protein of Bcl-2 family proteins member, which will form a channel in the mitochondria and causes the release of cytochrome c, eventually leading to apoptosis. Unlike Bax, Bcl-2 is an anti-apoptotic member of Bcl-2 family proteins; Bcl-2 inhibits the release of cytochrome c from the mitochondria, thus inhibiting apoptosis (Adams and Cory, 2018). If our treatment has an effect on apoptosis, we should notice a decrease in the amounts of full length PARP-1 and caspase-3 and also in Bcl-2 and an increase in the amount of Bax. For full length PARP-1 and full-length caspase 3 and Bcl-2 proteins the results showed a significant decrease in their expression in A549 cells treated with *C. aronia* L. for 48 hr (Figure 7A, B). The level of Bax protein was unaltered (Figure7). These results indicated that *C. aronia* L. induced apoptosis through PARP-1, caspase-3 and Bcl-2.

*Detection of apoptosis induced by C. aronia* *L. by flow cytometry assay*

**Figure 8:**
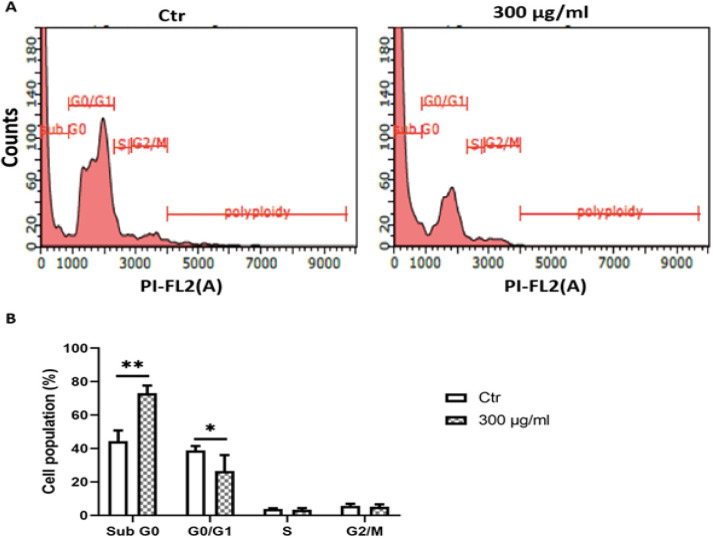
Effect of *C. aronia* L. on the Cell Cycle Phase Distribution. (A) DNA content of A549 cells treated with *C. aronia* L. (300 µg/ml) for 48 hr was analysed by propidium iodide (PI) staining. The cell cycle distribution is quantitatively measured by using flow cytometry. (B) The values of the histogram indicate the percentage of cells (before and after treatment) in the indicated phases of the cell cycle. Data are presented as the mean ± SEM for three independent experiment

Western blot results indicate that the *C. aronia* L. induce apoptosis. To confirm these results, we also observed the induction of apoptosis via a propidium iodide (PI)/RNase A Staining Buffer which is analyzed by flow cytometry. As shown in Figure 8, flow cytometry analysis indicates that the treatment of A549 cells with 300 µg/ml of *C. aronia* L. extract for 48 hr induces apoptosis by increasing the percentage of A549 cells in sub G0 (from 44.31.8% to 73.04%) and by decreasing the percentage of A549 cells in G0/G1 (from 38.87% to 26.48%) compared to control A549 cells exposed only to DMSO (Dimethyl Sulfoxide). Since no significant change is observed in the cell population of S phase or G2/M phase, it is suggested that *C. aronia* L. do not exhibit a cytostatic activity.

## Discussion

In the last few years, cancer has become the second cause of death worldwide (Bray et al., 2018). The high rates of deaths caused by cancer referred to the difficulty of its diagnosis in the early stages, excluding breast cancer, in addition to the high speed of cancer progression, metastasis and resistance to treatments (Al-Azri, 2016). Lung cancer and especially the non-small cell lung adenocarcinoma is considered the most abundant cancer worldwide for both males and females (de Groot et al., 2018). The high risks of lung cancer can usually be caused by industrial pollution, by life style and mostly by smoking including passive smoking (Al-Azri, 2016).

Based on the stage of lung cancer, chemotherapy, radiotherapy and surgery are the main lung cancer treatments. Since chemotherapy and radiotherapy with their side effects are not enough to fight cancer, the actual medicine returns to traditional herbal medicine to find plants with high treatment efficiency and with low side effects which can increase the survival rate of a patient with cancer. 

*Crataegus *genus is well known as hawthorns that belong to the Rosaceae family. This genus is mainly distributed in the Northern hemisphere all over Asia, Europe and North Africa. The Crataegus genus has approximately 1,000 species identified worldwide (Liu et al., 2010; Wen et al., 2015). The main focus of this study was on the *Crataegus aronia* hawthorn species that dominates the Arab countries. Besides its ornamental uses, *C. aronia* has been utilized in traditional medicine in treating of many disorders such as diabetes, hyperglycemia, cardiovascular diseases (CVD), cancer and sexual weakness (Ali-Shtayeh et al., 2000; Ljubunic et al., 2005; Al-Hallaq et al., 2013). However, despite its wide use in traditional medicine, there are a few reports in the literature displaying the anti-cancer effects of *C. aronia.*

We begin our study by evaluating the antioxidant activity of the hydroalcoholic extract of *C. aronia* (leaves, fruits, seeds) because it is widely highlighted in literature that the compound with strong antioxidant property will, in general, have potential anticancer effect since the free radicals have a role in cancer development (Kaur and Kapoor, 2001). A study shows that the water extract of leaves and fruits of *C. aronia* have a significant antioxidant activity (Nabavi et al., 2015). Our data indicate that hydroalcoholic extract of *C. aronia* leaves have the highest antioxidant activity. A study was done to evaluate the effect of hydroalcoholic extract of *C. aronia* on human liver cancer cell line (HepG2) by MTT assay. The results have shown that the *C. aronia* has a significant effect on HepG2 just with high concentration 500 μg/ml after 24 and 72 hr of treatments (Kmail et al., 2015). Another study on the ethyl acetate extract of *C. aronia* has shown that it inhibited both human colorectal cancer cell lines HT-29 and HCT-116 cell lines proliferation in a dose dependent manner (Mustapha et al., 2016). To prove the anticancer effect of hydroalcoholic extract of *C. aronia,* we have studied the anti-proliferative activity of *C. aronia* (leaves, fruits, seeds) to show that if there is a correlation between antioxidant and antiproliferative of cancer cells. Cell viability assay was carried out using MTT assay in human lung cancer cell line (A549), like antioxidant, our results show that the *C. aronia* L. have the highest inhibition of viability on A549 cell line. This intense antiproliferative and antioxidant activity of *C. aronia* could be attributed to phenolic compounds presence (Šavikin et al., 2014). Here, our results show that *C. aronia* may have future therapeutic implications by protecting cells against oxidative stress.

According to the results obtained, the leaves of *C. aronia, *which have the highest antioxidant and anti-proliferative activity, were chosen for further experiments. For cancer treatment, researches have been conducted to combine chemotherapy with plant extracts to decrease the side effects and boost the anticancerous treatment. Cisplatin is one of the most compelling ones between many numerous platinum based-chemotherapy drugs used for lung cancer treatment (Hotta et al., 2004). In this work, we treated the A549 cells for 48 hr with cisplatin, *C. aronia* L. and combination cisplatin/*C. aronia* leaves. The results show that the *C. aronia* L. boosted the activity of the low concentration of cisplatin, so the *C. aronia* L. can be used in combination with cisplatin to treat lung cancer with fewer side effects and offer more effective health benefits than higher-dose of cisplatin. 

Chemotherapy is usually performed via intravenous routes which causes hemolysis for the human red blood cells (RBCs). Our data indicated that the percentage of inhibition of hemolysis has been greatly increased by the *C. aronia* leaves. These results make it possible to use the *C. aronia* L. for the treatment of lung cancers by intravenous injection.

Cancer cell migration (metastasis) in patients is a major cause of cancer-related mortality (Talmadge and Fidler, 2010; Zheng et al., 2018). Hence, inhibiting migration is an essential step for the treatment of cancer. Despite progress in research, there are until now limited effective therapies available against the metastatic of lung cancer due to drug resistance and severe side effects (Dasari and Tcgounwou, 2014). For this purpose, we investigated the impact of *C. aronia* L. on the migration ability of A549 cells by using wound-healing assay. We performed this test with concentrations of plant extracts and times of treatment that were non-cytotoxic to A549. The toxicity of treatments was evaluated by MTT assay. The present study demonstrated that *C. aronia* L. suppressed the migration in-concentration and time-dependent manner. In addition, when cancer cells undergo metastasis, they lose their ability to adhere to each other so they can invade other organs of the body. When cancer is treated, the adhesion of cells (aggregation) should increase so the metastasis decrease (Abduljauwad and Ahmed, 2019). To identify if our extract has an effect on the cell-cell adhesion of A549 cells, aggregation assay was performed. The number of single cells in control (without treatment) and in treatment with *C. aronia *L. was counted and the percentage of aggregation was calculated and compared to the control. The results of the present study show that the number of single cells decreased and the cell aggregation percentage increased when cells were treated with *C. aronia *leaves. Taken together, our results demonstrated that the *C. aronia *L. can be used as a potential treatment for lung cancer metastasis owing to its anti-metastatic, antiproliferative and therapeutic effects. Because our data clearly show that hydroalcoholic extract of *C. aronia *L. decreases the viability of A549 cells and because several literatures indicate that natural compounds derived from plants promote cancer cell death through apoptosis induction (Safarzadeh et al., 2014), we have investigated the effect of *C. aronia* L. on apoptosis signaling pathway where there is no study about it. The process of apoptosis occurs normally during the lifetime of an organism, which is regulated by group of proteins. The most proteins that are involved in apoptosis are the Bcl-2 family proteins, falling into pro-apoptotic proteins such as Bax, Bim and Bid and anti-apoptotic proteins such as Bcl-2, Bcl-XL and Mcl-1 (Shamas-Din et al., 2013). Many other factors identified were shown to play a vital role in apoptosis, such as p53, caspase-3 and PARP-1. P53 is a tumor suppressor protein that prevents cell cycle progression and induces apoptosis. P53 is the most studied gene in cancer biology since it is mutated in most types of cancers including lung cancer. When DNA is damaged in the cell, p53 expression will rise, leading to an increase in Bax protein expression, which is a pro-apoptotic protein of Bcl-2 family proteins member, which will form a channel in the mitochondria leading to the release of cytochrome c, eventually leading to apoptosis (Chipuk et al., 2004). Unlike Bax, Bcl-2 is an anti-apoptotic member of Bcl-2 family proteins that prevents the release of cytochrome c from the mitochondria, which will consequently inhibit apoptosis (Papaliagkas et al., 2007).

The activation of caspase-3 is a central event in the process of apoptosis (Wolf and Green, 1999; Adams and Cory, 2018). The caspase-3 is activated by cleavage. Poly (ADP-ribose) polymerase-1 (PARP-1) functions in the repair of DNA damage by adding poly (ADP ribose) polymers in response to a variety of cellular stresses (Chaitanya et al., 2010).

During the execution phase of apoptosis, pro-caspase 8 is cleaved and is activated, which will then cleave and activate caspase 3. Cleaved caspase 3 will then cleave PARP-1 (Thornberry and Lazebnik, 1998). Western blot data indicated that Bcl-2 protein expression levels, full length PARP-1 and full-length caspase-3 decreased in A549 cells treated for 48 hr by hydroalcoholic extract of *C. aronia *L. To confirm the results of the western blot, the effect of *C. aronia *L. on cell cycle distribution was tested by flow cytometry analysis. Treatments that modify the cell cycle reflect the cellular mechanism caused by these treatments, such as apoptosis, necrosis, proliferation, differentiation and cell cycle arrest (Elmore, 2007). The increase of cells in sub G0 was taken to reflect the induction of apoptosis (Thangaraj et al., 2019). The flow cytometry results showed that hydroalcoholic extract of *C. aronia *L., by comparing to control, has apoptosis activity on A549 cells by increasing the percentage of cells in sub G0 phase and by reducing the percentage in G0/G1 phase. These results led us to draw a conclusion that ethanol water extract of *C. aronia* L. decrease the viability of lung cancer cells A549 by apoptosis. All our results encouraged us to evaluate in the future the profile of polyphenols in the hydroalcoholic extract of *C. aronia* leaves that may be responsible for the demonstrated activities. 

In conclusion, this study is the first to show that hydroalcoholic extract of *C. aronia* L. can decrease the viability of lung cancer cells A549 by causing apoptosis, which was associated with decrease levels of Bcl-2, full length PARP-1 and full-length caspase-3 proteins. Furthermore, *C. aronia* L. were able to suppress the A549 migration by decreasing wound closure properties and by increasing a cell-cell aggregation. Results from this work suggest that *C. aronia* L. could be a new natural effective candidate for the treatment of the lung cancer.

## Authors’ contributions

IO performed the biological experiments, FK analysed the results and corrected the manuscript, SN performed the plant collection and extraction, conceptualized the study, analysed the results and drafted the manuscript. All authors read and approved the final manuscript.

## Abbreviations

*C. aronia* L.: *C. aronia* leaves; SCLC: Small Cell Lung Cancer; NSCLC: Non-Small Cell Lung Cancer; DMEM: Dulbecco’s Modified Eagle’s Medium; FBS: Fetal Bovine Serum; PBS: Phosphate-Buffered Saline; PI: Propidium Iodide; OD: Optical Density; RBCs: Red Blood Cells; DMSO: Dimethyl Sulfoxide.

## Summary

The studies showed that *C. aronia* L. had potent anti-oxidant activities. The leaves extract had a protective effect against human red blood cells hemolysis and showed beneficial effects against human lung cancer cell line (A549) by reducing wound healing migration, by increasing cell aggregation and by exhibition of apoptotic activity.
